# Synergetic improvements of sensitivity and specificity of nanowire field effect transistor gene chip by designing neutralized DNA as probe

**DOI:** 10.1038/s41598-018-30996-4

**Published:** 2018-08-22

**Authors:** Wen-Pin Hu, Chih-Chin Tsai, Yuh-Shyong Yang, Hardy Wai-Hong Chan, Wen-Yih Chen

**Affiliations:** 1 Department of Bioinformatics and Medical Engineering, Asia University, Taichung, 41354 Taiwan; 2 Department of Chemical and Materials Engineering, National Central University, Jhong-Li, 32001 Taiwan; 3 Institute of Biological Science and Technology, National Chiao Tung University, Hsinchu, 30010 Taiwan; 4 Helios Bioelectronics, Inc. 3F., No.2, Sec. 2, Shengyi Rd., Zhubei City, Hsinchu County 302 Taiwan

## Abstract

Neutral DNA analogs as probes for the detection of target oligomers on the biosensors based on the field-effect transistor (FET) configuration feature advantages in the enhancement of sensitivity and signal-to-noise ratio. Herein, we used phosphate-methylated nucleotides to synthesize two partially neutralized chimeric DNA products and a fully neutralized DNA sequence and adopted a regular DNA oligomer as probes on the polycrystalline silicon nanowire (NW) FET devices. The sequences of two neutralized chimeric DNAs close to the 5′ end were alternately modified with the phosphate-methylated nucleotides, and all probes were immobilized via their 5′ end on the NW surface. The non-specific-to-specific binding ratio indicated that the two 5′-end partially neutralized chimeric DNAs featured better performance than the regular and fully neutralized DNA oligomers. The partially neutralized probe design reduces the ionic strength needed for hybridization and increases the Debye length of detection, thus promoting the detection sensitivity of FET and achieving the limit of detection of 0.1 fM. By using an appropriate probe design, applying DNA oligomers with embedded phosphate-methylated nucleotides in the FET biosensors is a promising way for gene detection with high sensitivity and specificity.

## Introduction

The silicon nanowire (NW) field-effect transistor (FET), as a biosensor, promises ultra-high sensitivity in the detection of a variety of proteins^1^, nucleic acids^2–4^, and metal cations^5^. Owing to the advantages of real-time, label-free, selectivity, and sensitivity detection capabilities, the FET-based detection technique continually receives significant attention. Biological or chemical molecules linked to the chemically functionalized NW surfaces render the FET chips with capabilities for sensing target molecules. The variations in electrical properties of NW in biosensing depend on the charges carried by the captured molecules upon biomolecular binding. NW biosensors are also very sensitive to pH conditions, which are related with protonation and deprotonation of surface hydroxyl groups on the SiO_2_ surface at the solution–dielectric interface^6^. The ion-sensitive FET causes remarkable changes in the detection of DNA hybridization. In the field of nucleic acid sensing, the single-stranded DNA molecule is usually immobilized on the NW surface as the probe/receptor for the detection of its complementary strand. Nucleic acid hybridization is a highly specific interaction, and the increase in the amount of negative charge at the gate surface transduces into an electrical signal that reflects the result of hybridization. However, the phosphate groups, such as DNA and RNA, on the backbone of nucleic acid molecules cause these fragments to carry highly negative charges in physiological conditions. By reason of high-negative charges, electrostatic repulsion exists between the probe and target sequences, decreasing the hybridization efficiency. The ionic screening effect (known as Debye length) and the background noise generated by salt ions in the buffer also critically influence the measurements of Si NW-FETs. The movement of bound DNA or RNA probes on the FET chip was also discovered to generate high background noise^6^.

Considerable efforts have been dedicated to the development of DNA analogs owing to their unique properties, which are unfound in nature; DNA analogs can also hybridize specifically with natural target DNAs. These DNA analogs can form stable structures with target DNAs and improve the hybridization efficiency. Peptide nucleic acid (PNA), locked nucleic acid (LNA), phosphoramidate morpholino (MORF), and hexitol nucleic acid oligomers have been employed in therapeutic applications; these DNA analogs exhibit better biostability in body than regular nucleic acids without compromising the specific ability to the complementary target nucleic acids^7^. The neutrally charged PNA can significantly improve DNA-binding efficiency, and the PNA used as a capture probe/receptor in the FET-based biosensor can achieve ultrasensitive label-free detection of nucleic acid fragments^7,8^. The PNA-functionalized FET biosensor can be operated for the detection of complementary DNA fragments in low-ionic-strength environment with a high signal-to-noise ratio (SNR). LNA oligomers are synthesized chemically, whereas DNA or RNA residues can be mixed in the oligonucleotide to produce a chimeric oligomer. LNA is also used for the development of high-performance affinity and specificity biosensors in the detection of natural nucleic acids, especially microRNAs^9^. The nDNA oligomer composed of neutral nucleotides are used as probes for the detection of target nucleic acid fragments^4^. Higher degree of probe immobilization, improved hybridization efficiency, and the prevention of charge interference from the probe are the virtues of using nDNA oligomers in the FET measurement for the detection of oligonucleotides^4^. Other uncharged analogs, such as MORF oligomers and methylphosphonate oligonucleotides, are synthesized and mainly applied in therapeutic developments^10,11^ and diagnostics^12^.

The neutral character of DNA analogs allows them to avoid the interferences in the binding buffer and binding reactions from the background electric charges in FET measurements, resulting in the improvement of sensitivity in DNA detection^13^. The properties of uncharged DNA analogs also show potential in the applications of FET-based biosensors due to their stability and binding capabilities to targets in low-ionic-strength conditions. Herein, we compared the performances of using regular, fully neutralized, and partially neutralized chimeric DNA probes on the FET device in buffer solutions with different salt concentrations. This study also evaluated the specificity and sensitivity for each type of probes in the detection of target fragment. The target fragment, cH1, originates from hemagglutinin 1 DNA in influenza virus strains, and it is highly associated with diverse genetic diseases. The partially neutralized chimeric DNAs were modified with phosphate-methylated nucleotides close to the 5′ end to reduce the charge interference from the probe within the sensing range of the FET device and to maintain specificity. The limit of detection (LOD) for the FET device coupled with partially neutralized chimeric DNAs was investigated. To the best of our knowledge, this study is the first to report the novel probes designed for use in FET-based biosensors. Consequently, we expected that the partially neutralized chimeric DNA probes could further improve the performance of the FET sensors in the measurement of target DNA to provide high-sensitive and high-accuracy detection results.

## Materials and Methods

### Reagents and DNA fragments

3-Aminopropyltriethoxysilane (APTES), bis-tris propane (BTP), sodium cyanoborohydride (NaBH_3_CN), and glutaraldehyde (GA) were bought from Sigma–Aldrich (USA). Ethanol (99.5%) and acetone were purchased from Echo Chemical Co., Ltd (Taiwan). All the BTP buffer solutions with different concentrations (1, 10, and 100 mM) and were used in the polycrystalline silicon (poly-Si) NW-FET measurements were prepared in deionized water and adjusted to pH 7.0. Deionized water was purified by an ultra-pure water system (18.2 MΩ·cm, Millipore, USA). All other chemicals used in this study were of reagent grade.

The four probe DNAs (H1-DNA, H1-nDNA, H1-nDNAp5, and H1-nDNAp4) all featured the same sequences, but the structures of phosphate-methylated nucleotides in the fully neutralized and partially neutralized chimeric DNAs differed from that of general nucleotides. Table 1 shows the differences between the probe DNAs; the superscript “n” denotes the phosphate-methylated nucleotides. Fully neutralized and partially neutralized chimeric DNAs were synthesized and supplied by Helios Bioelectronics Inc. (Hsinchu, Taiwan). The target DNA and probe H1-DNA oligomers were purchased from MDBio Inc. (Taiwan). For the synthesis of DNA analog with phosphate-methylated phosphate groups, Fmoc-protected phosphoramidites were used to synthesize fully neutralized and partially neutralized chimeric DNAs. The neutralized DNA analog used in this study possessed the “RO–P–O” backbone (where R is the methyl group). The fully neutralized probe H1-nDNA features 18 phosphate-methylated nucleotides in the backbone. The partially neutralized probes H1-nDNAp5 and H1-nDNAp4 contain five and four phosphate-methylated nucleotides in the backbone, respectively. The target DNA, cH1, is a perfect-matched target for the probe H1-DNA. On the other hand, the target ncH1 is non-complementary to the probe H1-DNA, which is designed to test the signal caused by non-specific adsorption on the biosensor platform. The characteristics of H1-nDNAp4/cH1 duplex were revealed and proven by circular dichroism spectroscopy in a previous report^14^. Notably, the duplexes with and without methyl phosphonate backbone showed no significant differences in their secondary structures^14^.

**Table 1:** Sequences of regular DNA, neutralized DNA, neutralized chimeric DNA, and target DNA molecules.

DNA	Identifier	Sequence (5′ to 3′)
Probe	H1-DNA	NH_2_-C_6_-CACACTCTGTCAACCTAC
	H1-nDNA	NH_2_-C_6_-C^n^A^n^C^n^A^n^C^n^T^n^C^n^T^n^G^n^T^n^C^n^A^n^A^n^C^n^C^n^T^n^A^n^C^n^
	H1-nDNAp5	NH_2_-C_6_-C^n^AC^n^AC^n^TC^n^TG^n^TCAACCTAC
	H1-nDNAp4	NH_2_-C_6_-C^n^ACA^n^CTC^n^TGT^n^CAACCTAC
Target	cH1	CCATTGTGACTGTCCTCAA GTAGGTTGACAGAGTGTG
	ncH1	TGATAACCAATGCAGATTTG

The superscript “n” denotes the neutralized nucleotides with phosphate-methylated phosphate groups.

### Poly-Si NWFET sensor

In this study, the poly-Si NWFET sensor used was fabricated by the National Nano Device Laboratories (Hsinchu, Taiwan) based on the design of Prof. Yang’s group at the National Chiao Tung University (Hsinchu, Taiwan). The detailed fabrication process of poly-Si NWFET sensors was reported in a previous research^1^. The poly-Si NWs of an n-type FET were prepared using the sidewall spacer formation technique. For each poly-Si NW-channel, the length and width of NW measured 2 μm and 80 nm, respectively. The current–voltage (I–V) characteristics of poly-Si NWFET sensor were measured by using the Keithley 2636 A Dual-Channel System SourceMeter instrument (Tektronix, Inc., USA). In addition to Keithley 2636 A, a probe station with a chamber (EverBeing Int’l Corp., Taiwan), a microfluidic system composed of a polydimethylsiloxane flow cell, an acrylic gasket and metal plates, and a programming syringe pump (KD Scientific) were all used in the measurement. The microfluidic system was integrated with the Si NW-FET sensor (shown in Supplementary Fig. S1). The dimension of fluidic channel was 5 × 0.5 × 0.1 mm^3^. The programming syringe pump transported the liquids continuously through the NWFET sensor at a flow rate of 83 μl/min. In the measurement of drain I–V (*I*_d_−*V*_g_) curve, the drain voltage (*V*_d_) was set at 0.5 volt, whereas the gate voltage was swept from −1 V to 2 V with a sweep interval of 0.2 V. Before carrying out the experiments, the *I*_d_−*V*_g_ curves were measured in triplicate at room temperature after incubation for 4 min in the BTP buffer to ensure the stable signal of the poly-Si NWFET sensor. Afterward, this *I*_d_−*V*_g_ curve was used as the baseline to evaluate the shifts in electric properties caused by the following biorecognition events. Then, the buffers containing target DNAs were initially injected through the chip surfaces for 10 min, and the syringe pump was stopped for 30 min to ensure better hybridization reactions of the target nucleotide sequences with the immobilized probes. To eliminate non-specific binding to the probe or to the chip surface, the BTP buffer was subsequently pumped into the flow channel for 10 min. After the washing process, the final *I*_d_−*V*_g_ curve was recorded until the same *I*_d_−*V*_g_ curve was repeatedly measured more than three times.

For the quantitative analysis of experimental results, the data of *I*_d_−*V*_g_ curves before and after hybridization were used to calculate the change in gate voltage (ΔV) at a drain current (*I*_d_) of 1 × 10^−9^ A. The current magnitude of 1 × 10^−9^ A is suitable for the quantitative analysis of gate voltage change because it is approximately the middle of the measured current range. The change in gate voltage (ΔV) was calculated as follows:

1 $${\rm{\Delta }}V={V}_{d1}-{V}_{d0}$$

where *V*_*d1*_ refers to the gate voltage for the electric response generated after hybridization at *I*_d_ equal to 1×10^−9^ A. Herein, *V*_*d0*_ denotes the gate voltage for the electric response of NW surface with the immobilized probes when *I*_d_ equals 1×10^−9^ A.

In addition, blank experiments were designed to test the stability of FET devices used in the measurements. *I*_d_−*V*_g_ curves were measured in triplicate for each chip at room temperature after incubation for 4 min in the BTP buffer to make sure the signals of the poly-Si NWFET sensor were stable. Until the three *I*_d_−*V*_g_ curves were stable, we recorded the *I*_d_−*V*_g_ curve as the initial baseline. The voltage changes between the initial baseline and final voltage at *I*_*d*_ of 1×10^−9^ A was used to evaluate the stability of the device.

### Probe immobilization on the sensor surface

Initially, the poly-Si NWFET was immersed in acetone and sonicated for 10 min to remove the contaminants adhering to the surface. Subsequently, we used ethanol to perform the immersion and sonication cleaning processes again for 10 min. In the last cleaning procedure, deionized water was utilized to clean the chip in the ultrasonic cleaner bath by using the same steps as described above. After the cleaning procedures, we used a plasma cleaner (PDC-32G, Harrick Plasma, USA) to treat the chip for cleaning and producing OH radicals on the surface^15,16^. The OH radical benefited later chemical modifications. Then, we immersed the chip in APTES (2% in ethanol) for 30 min at room temperature. The APTES could link to OH groups and present NH_2_ groups on the surface. The NWFET chip was then immersed in ethanol and sonicated for 10 min. Afterward, the chip was placed on the heating plate at 120 °C for 10 min. Next, the GA solution (12.5% in 10 mM sodium phosphate buffer (Na–PB) was used to soak the chip for 1 h in a dark container to functionalize the NW surface with numerous aldehyde (-CHO) groups. The FET chip was cleaned thrice with the Na–PB buffer and blown dry with nitrogen.

To immobilize the probes on the chip surface, 10 mM sodium phosphate buffer containing 1 µM probe DNA (regular, fully neutralized, or partially neutralized chimeric DNA) was prepared to spot on the NW by using a microarray spotter (SmartArrayer 136, CapitalBio Corporation, China) and incubated with the FET chip overnight. Each kind of probe DNA possessed an amino group at the 5′ end of sequence. After the overnight immersion, we used the Na–PB buffer to wash the chip thrice and utilized 4 mM sodium cyanoborohydride (in 10 mM Tris buffer) to immerse the chip for 30 min. Cyanoborohydride can react with the immobilized probes and stabilize the CN groups on the probe backbones. Tris buffer can de-activate or block the remaining active carbonyl (-CHO) groups to prevent non-specific binding on the NW surface.

## Results and Discussion

### Comparison of the ratio of non-specific binding to specific binding of each probe

**Figure 1:**
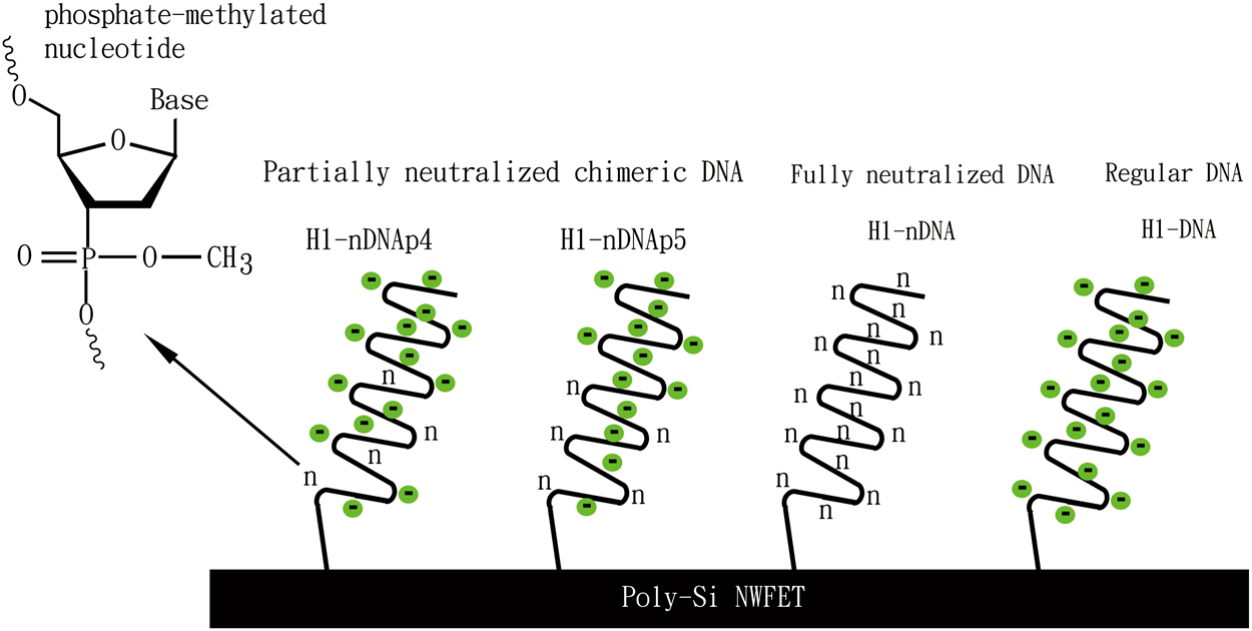
Schematic illustration of four DNA probes used in FET measurements. Phosphate-methylated nucleotides are denoted by “n”.

**Figure 2:**
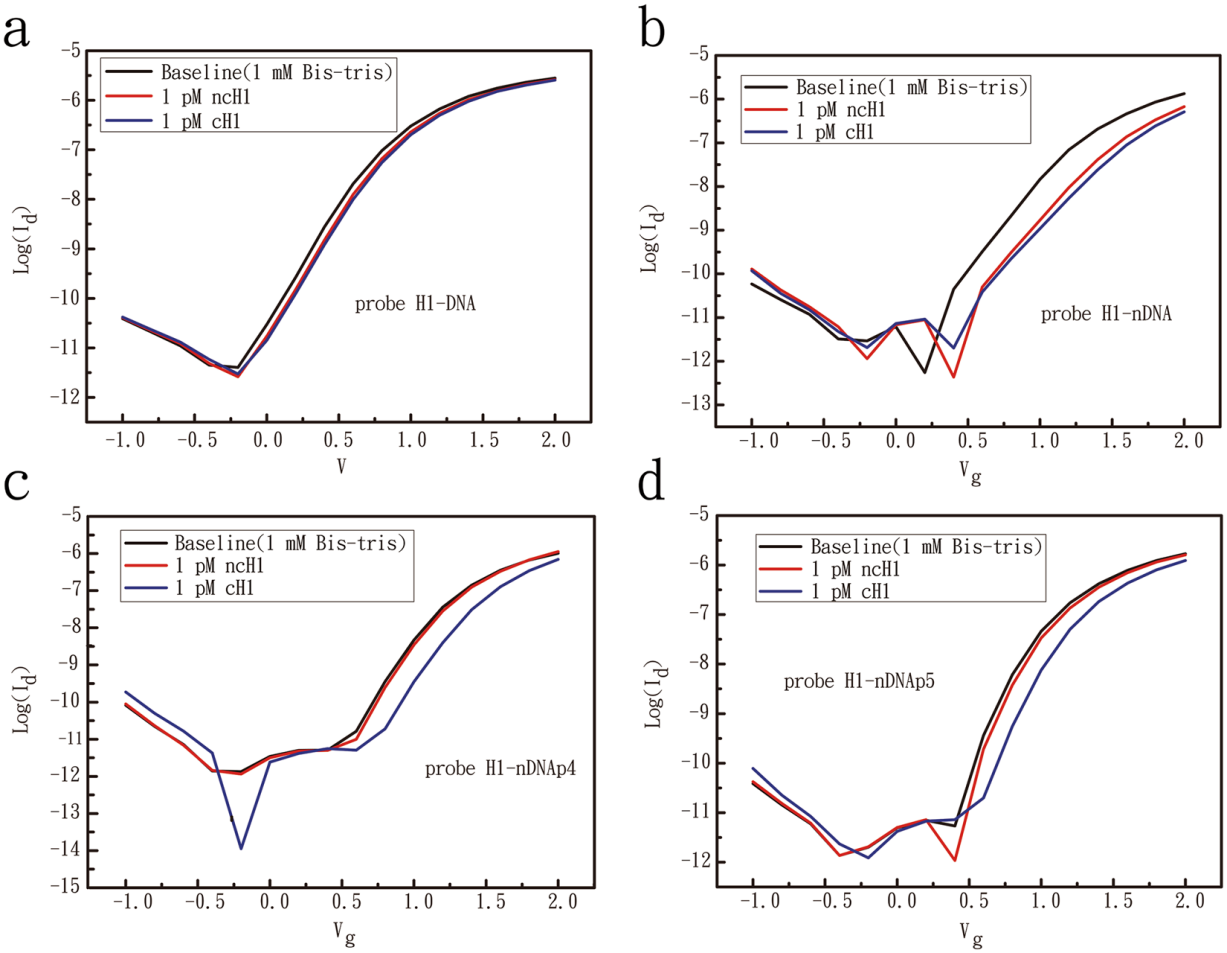
Experiments performed on poly-Si NWFET devices for testing the DNA-binding specificity of probe H1-DNA (**a**), H1-nDNA (**b**), H1-nDNAp4 (**c**), and H1-nDNAp5 (**d**) oligomers. The concentration of complementary (cH1) and non-complementary (ncH1) target sequences was 1 pM, and experimental results were measured in 1 mM BTP.

Initially, we compared the differences between the hybridization results of using regular and fully neutralized DNA probes in the measurement of perfect-matched (cH1) target DNA fragment and non-complementary target ncH1. The target DNA samples, including cH1 and ncH1, were all prepared at 1 pM in 1 mM BTP. According to published reports^17,18^, several cations in the buffer could cause gate bias instability problems in the FET devices. To eliminate these instability factors, we adopted BTP in the FET measurements without adding salt. Because salt ions in the hybridization buffer can reduce the FET Debye length and therefore decrease the detection sensitivity. The low-ionic-strength BTP is an organic buffer that contains trace amounts of metal ions and can replace conventional mineral buffers in various biological applications. BTP is also a kind of zwitterionic buffer possessing both positive and negative charges and features low interference with biological reactions. Figure 1 shows the four nucleic acid probes used in the FET measurements; Table 1 shows the detailed sequence data. Figure 2 presents the representative I–V curves for the probe–target hybridizations. The ratio of non-specific binding to specific binding (α) of each probe is calculated by using Equation (2):

2 $${\rm{\alpha }}=\frac{{\rm{\Delta }}{V}_{h}}{{\rm{\Delta }}{V}_{ncH1}}$$

where Δ*V*_*h*_ is the gate voltage change at the electric current of 1 × 10^−9^ A before and after the hybridization of complementary sequence (cH1). Δ*V*_*ncH1*_ represents the gate voltage change at the electric current of 1 × 10^−9^ A before and after the injection of target ncH1 for hybridization.

As shown in Fig. 2a, the α value of regular DNA probe is remarkably low (α = 1.36), whereas the fully neutralized probe H1-nDNA features an α value of 1.23 (Fig. 2b). The two partially neutralized chimeric DNAs (H1-nDNAp4 and H1-nDNAp5) were designed and applied in the measurements. These two partially neutralized chimeric DNAs exhibited good performance in the improvement of non-specific binding, especially for probe H1-nDNAp4. The α values for probe H1-nDNAp4 (Fig. 2c) and H1- nDNAp5 (Fig. 2d) reached 7.95 and 4.43, respectively. The online Supplementary Table S1 summarizes the data on gate voltage changes and α values for the probe–target hybridizations. Compared with H1-DNA, the experimental data also prove that two partially neutralized chimeric DNAs can effectively enhance the signals generated from the specific binding on the FET device.

Diercks *et al*.^19^ observed that LNA probes exhibited no improved performance in signal enhancement compared with the conventional DNA probes on the biosensor. In addition, the LNA probes showed an enhanced sensitivity in microarray applications. However, more false-positive signals were obtained from these measurements. They speculated that LNA probes utilized under certain solid surface-hybridization applications could result in worse sensitivity, specificity, and stability. However, the neutral probe could provide better efficiency in hybridization and enhance the signal on biosensors^4^. In our study, the probe H1-nDNA with the uncharged backbone could contribute to the signal enhancement of DNA hybridization on the FET device but also increase the non-specific binding response. By using two partially neutralized chimeric DNAs as probes, non-specific binding could be efficiently reduced and retain the effect of signal enhancement on the FET device. We supposed that the negative-charged backbones of two partially neutralized chimeric DNAs could provide appropriate repulsion force to resist non-specific binding. The probe design achieves a balance in improving hybridization efficiency and reducing specificity.

### Effect of ionic strength on hybridization

**Figure 3:**
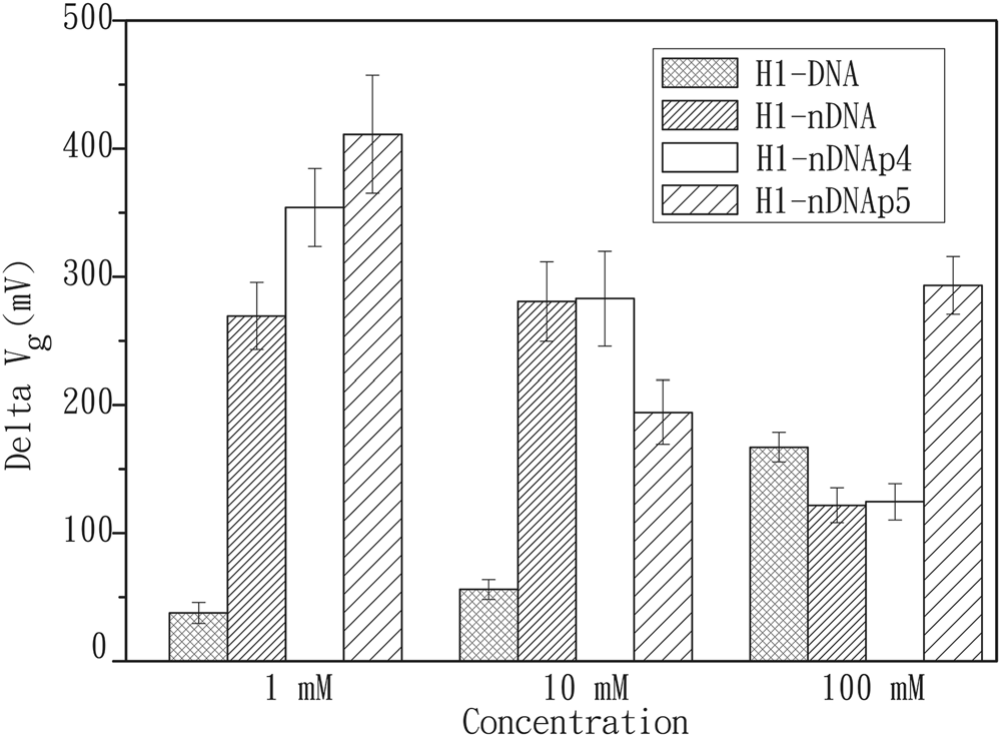
Gate voltage changes measured in 1, 10, and 100 mM BTP buffers. The signals were produced by the formations of H1-DNA/cH1, H1-nDNA/cH1 H1-nDNAp4/cH1, and H1-nDNAp5/cH1 duplexes. Target DNAs, cH1, and ncH1 were all prepared at 1 pM.

To compare the measured signals in the buffers of different concentrations, four probes were immobilized on the NW surface to interact with the target cH1 in 1, 10, and 100 mM BTP buffers (Fig. 3; representative I–V curves can be found as Supplementary Figs S2a–e and S3a–e). Probe H1-DNA is a regular DNA. Therefore, the gate voltage change raises with the increase in buffer concentration. A regular DNA can associate its complementary fragment easily at a high concentration condition (with more charges in the buffer) because screening of the negatively charged phosphate groups in the backbone reduces the electrostatic repulsion. On the contrary, the gate voltage changes for the other oligonucleotide probes containing phosphate-methylated nucleotides decreased with increasing buffer concentration. Except for probe H1-nDNAp5, the amount of gate voltage change in the 100 mM BTP buffer is higher than that in 10 mM BTP buffer.

These results suggest the two major effects involved in hybridization experiments: (1) the probe’s ability to hybridize to the target; (2) the sensitive dependence of the Debye length. Hybridization is affected by the electrostatic repulsion between two complementary DNA nucleotides. For the nucleotide probes of H1-nDNA, H1-nDNAp4, and H1-nDNAp5, the neutral backbone of phosphate-methylated nucleotide results in no electrostatic repulsion force between the complementary nucleotide in the probe H1-nDNA, and the phosphate-methylated nucleotides in two chimeric DNAs can reduce the repulsion force to the target. The Debye length features a longer sensing distance in the low-concentration (low-ionic-strength) buffer, providing better sensitivity for the FET device. The better hybridization effects provided by the reduced/no-charge probes and the longer Debye length contributed to high sensitivity measurements in 1 and 10 mM BTP buffers. With the increase in buffer concentration, Debye screening significantly influenced the readout of the FET device. Compared with the conditions in 1 and 10 mM BTP buffers, either H1-nDNA/cH1 or H1-nDNAp4/cH1 duplex in the 100 mM BTP generates a smaller but still significant gate voltage change. In these experiments, we noted that H1-nDNAp5 still showed favorable tendency for hybridization in the highest buffer concentration used in this study. We speculated that the hybridization capability of partially neutralized chimeric probe might not necessarily decrease with increasing concentration and could depend on the positions and numbers of methylated nucleotides designed in the immobilized end of the probe on the NW surface.

### LOD of neutralized and neutralized chimeric probes

**Figure 4:**
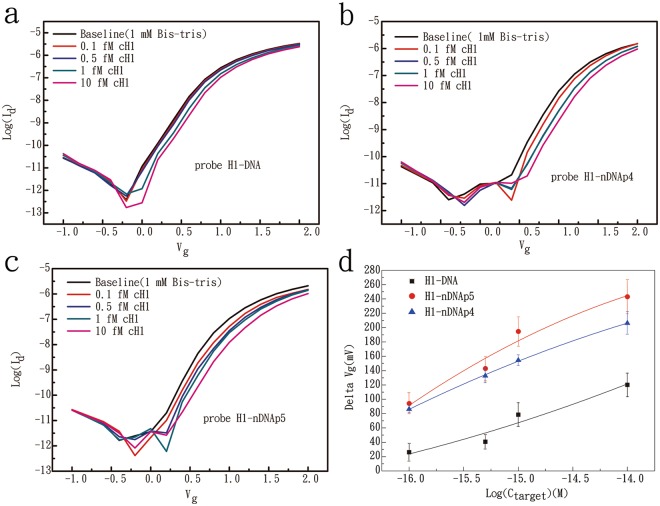
I–V curves for the detection of cH1 target molecules at concentrations of 10, 1, 0.5, and 0.1 fM with probes of H1-DNA (**a**), H1-nDNAp4 (**b**), and H1-nDNAp5 (**c**). (**d**) The calculated gate voltage changes of FET devices in the detection of complementary target oligomers with ultralow concentrations.

Four ultralow concentrations of target cH1 prepared (0.1, 0.5, 1, and 10 fM) in 1 mM BTP buffer were used to test the LOD of three different probes (H1-DNA, H1-nDNAp5, and H1-nDNAp4) on the FET device. We excluded probe H1-nDNA in this test due to its low specificity to the target oligomer. Initially, the buffer solution was injected into the flow channel, and the baseline of measurement was obtained until a stable I–V curve was repeatedly measured thrice. Then, the injection of target cH1 solution was started from 0.1 fM to 10 fM in sequence. For each probe, we used three FET chips to perform the hybridization experiments with the increasing target concentrations and obtain the data. After obtaining a stable I–V curve for each concentration of target solution, the target cH1 solution was changed. Figure 4a–c shows the I–V curves for the detection of cH1 target molecules at different concentrations with the use of three probes, and the experimental results are summarized in Supplementary Table S2. Probes H1-nDNAp4 and H1-nDNAp5 can produce 86–94 mV signal upon binding with the 0.1 fM target sequence. On the other hand, the regular DNA probe can generate a gate voltage change of 26 mV. The LOD of instrument is calculated based on the mean and standard deviation of the replicate blank readings, as presented in Equation (3):

3 $${S}_{dl}={S}_{reag}+3\times {\sigma }_{reag}$$

where S_dl_ refers to the signal at detection limit, S_reag_ denotes the signal for a reagent blank, and σ_reag_ is the known standard deviation for the reagent blank’s signal.

*S*_*reag*_ and *σ*_*reag*_ values were obtained from the data of three blank experiments performed by using different FET chips in which the initial (as the baseline), and final I–V curves were measured in 1 mM BTP buffer (see Supplementary Fig. S4 and Table S3). The data reveal the minimal gate voltage changes produced by noise in the three blanks, implying the stability of the device. The baseline was set as the initial point of the signal in each experiment. Therefore, the value of *S*_*reag*_ was 0. The standard deviation for the signals of triplicate reagent blanks calculated from experiments measured 19.6 mV, which could be considered as the background noise of electronic signal. The obtained LOD value of the instrument was 58.8 mV. The value of gate voltage change must reach higher than 58.8 mV. Otherwise, the gate voltage change was considered as the background noise.

Figure 4d shows the gate voltage changes and error bars in the detection of four ultralow concentrations of target cH1 on FET devices. The curves are fitted by the polynomial function. Notably, the regular DNA probe cannot precisely detect the target sequence of 0.1 or 0.5 fM because the value of signal is smaller than 58.8 mV. Nevertheless, probes H1-nDNAp4 and H1-nDNAp5 can detect the target sequence at as low as 0.1 fM, with the LOD reaching 0.1 fM. Compared with other studies, several Si NWFET nucleic acid sensors also presented femtomolar or lower LOD for the detection of DNA hybridization^6,20,21^. In this study, the two neutralized chimeric DNAs combined with the poly-Si NWFET devices provided comparable performances in the LOD to the level of 0.1 fM.

**Figure 5:**
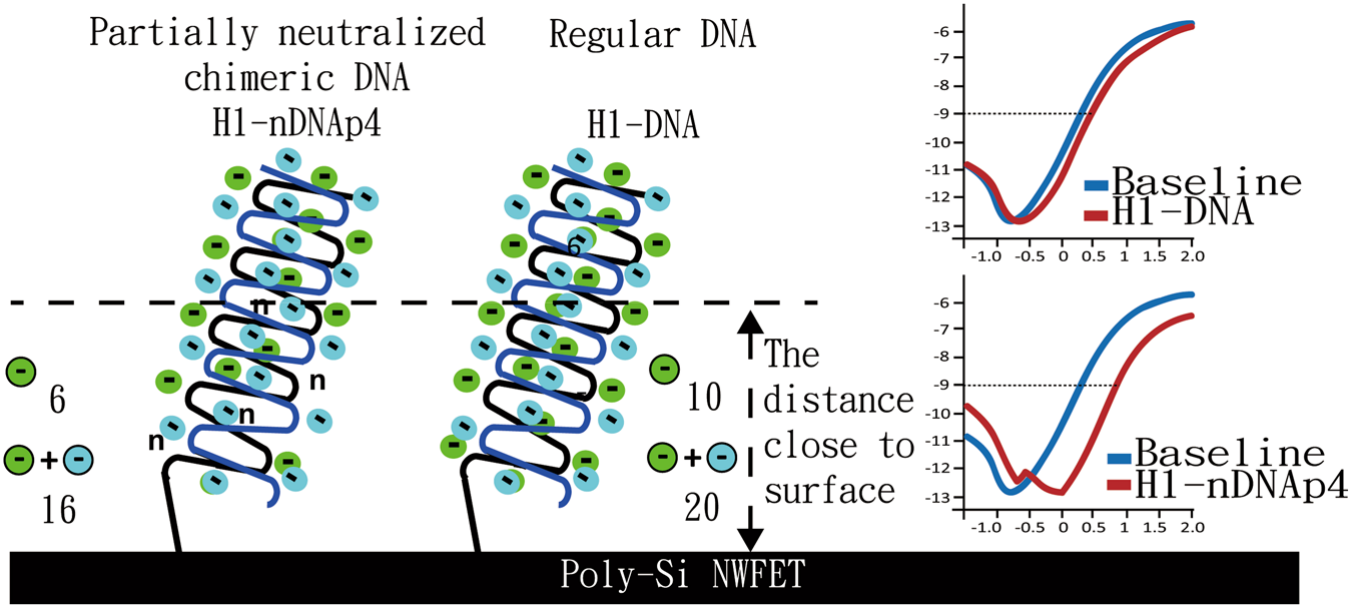
Charge variations adjacent to the surface of Si NW-FET chip before and after hybridization with the perfectly matched sequence. Small green and blue circles denote negative charges; the inserted *I*_*d*_*−V*_*g*_ curves at the right side present the performances of two different probes.

The FET is a charge-sensitive active electronic device; therefore, the charges carried by the probes are critical to the results in the FET measurement, similar to the improvement of sensitivity with the use of uncharged DNA analogue^13^. For biosensing purposes, charge screening phenomenon (Debye length) should be considered; on the other hand, the surface potential decays exponentially with distance to the electrode and electrolyte regions. At distances greater than the screening length, the charges of biomolecules are shielded by oppositely charged ions in a buffer solution. Therefore, variations in the surface charge density close to the NW surface can significantly contribute to the measured signals. According to experimental results, the two partially neutralized chimeric DNAs exhibited better sensitivity and specificity in the detection of target fragment, cH1, on the poly-Si NWFET sensors. By using the BTP buffer, the Debye screening length can be increased, and the length is sufficient to sense a part of the charge from the hybridized DNA complex. In addition, the concentration of BTP buffer is related to its capacity to provide appropriate charges to enhance the hybridization efficiency by reducing the intermolecular repulsion force. The enhancement of sensitivity of partially neutralized chimeric probe in FET measurements can be illustrated in a simple manner (Fig. 5). Concerning the charges carried by the 10 nucleotides of probe H1-nDNAp4 adjacent to the NW surface, the probe carries six negative charges (six phosphate groups). After hybridization, the target sequence brings 10 negative charges, whereas the duplex possesses 16 negative charges in this region. The negative charges close to the sensing surface increased by 2.67-fold, and the consequence then generated a larger conductance change on the poly-Si NWFET sensor. For probe H1-DNA, it just presented a 2-fold increase in the number of negative charges after hybridization. The partially neutralized chimeric DNAs demonstrated better hybridization capability with the target sequence especially in the buffer with low ionic strength^14^, which is another factor for producing significant conductance changes. The H1-nDNAp4/cH1 complex exhibited a higher melting point than the H1-nDNA/cH1 complex, and the hybridizations of complementary and non-complementary target DNAs with H1-nDNAp4 can be distinguished in the low-ionic-strength buffer at high operation temperature^14^. By altering the number and positions of embedded phosphate-methylated nucleotides in the design of probe, the melting point of probe/target complex can be adjusted. The detection strategy may also be applied in the FET measurement via manipulation of experimental temperature to enhance detection specificity and distinguish the perfect-matched sequence from single-base mismatched ones.

## Conclusions

We demonstrated the feasibility of enhancing FET sensitivity through using chimeric DNAs with methylated neutral nucleotides as probes. Remarkably, the positions of embedded methylated neutral nucleotides must be close to the end of the probe for immobilization on the sensing surface. The number and positions of embedded methylated neutral nucleotides are also critical to the performance of chimeric DNAs. These partially neutralized chimeric DNAs with novel design strategies are suitable for operation in buffers with low-ionic-strength, benefiting the sensitivity enhancement of FET-based measurement with high specificity. The partially neutralized chimeric probe DNAs shows a significant potential for the development of gene chips on the FET-based biosensors with high sensitivity and specificity.

## Electronic supplementary material

Supplementary Information
