# Supplementary material for: Synergetic improvements of sensitivity and specificity of nanowire field effect transistor gene chip by designing neutralized DNA as probe
Source: Sci Rep. 2018 Aug 22;8:12598. doi: 10.1038/s41598-018-30996-4 (PMC6105729; doi:10.1038/s41598-018-30996-4)
Supplement: Supplementary file 1 — Supplementary Information [file 41598_2018_30996_MOESM1_ESM.docx]

**Supplementary Information**

**Synergetic improvements of sensitivity and specificity of nanowire field effect transistor gene chip by designing neutralized DNA as probe**

Wen-Pin Hu^a,b^, Chih-Chin Tsai^c^, Yuh-Shyong Yang^d^, Hardy Wai-Hong Chan^e^, Wen-Yih Chen^c,^*

^a^ Department of Bioinformatics and Medical Engineering, Asia University, Taichung 41354, Taiwan

^b^ Department of Medical Laboratory Science and Biotechnology, China Medical University, Taichung City 40402, Taiwan

^c^ Department of Chemical and Materials Engineering, National Central University, Jhong-Li 32001, Taiwan

^c^ Institute of Biological Science and Technology, National Chiao Tung University, Hsinchu 30010, Taiwan

^e^ Helios Bioelectronics, Inc. 3F., No.2, Sec. 2, Shengyi Rd., Zhubei City, Hsinchu County 302, Taiwan

* Corresponding author. E-mail addresses: wychen@cc.ncu.edu.tw

CONTENTS

Table S1: The comparison of α values (ratio of non-specific binding to specific binding) for the probes in the detection of target fragment.

Table S2: The results of measuring different target-DNA concentrations by using three probes.

Table S3: Background signals in the blank experiments.

**Fig. S1:** Microfluidics System.

**Fig. S2:** Current-voltage curves obtained in 1, 10 and 100 mM Bis-tris propane (BTP) buffers with probe H1-DNA and H1-nDNA for the detection of target DNA.

**Fig. S3:** Current-voltage curves measured 1, 10 and 100 mM BTP buffers with probe H1-nDNAp4 and H1-nDNAp5 for the detection of target DNA.

**Fig. S4:** Three blank experiments performed by using different FET chips.

**Table S1.** Gate voltage changes and α values for the probe-target hybridizations in the 1 mM Bis-tris propane (α is defined as the ratio of non-specific binding to specific binding).

| **Identifier** | **Target cH1** | **Target ncH1** | **α value** |
| --- | --- | --- | --- |
| H1-DNA | 70 ± 8.5 mV | 51.4 ± 9.2 mV | 1.36 |
| H1-nDNA | 269.3 ± 19.7 mV | 218.1 ± 17.4 mV | 1.23 |
| H1-nDNAp4 | 205.1 ± 37.2mV | 25.8 ± 6.1 mV | 7.95 |
| H1-nDNAp5 | 173.3 ± 15.3mV | 39.1 ± 8.4 mV | 4.43 |

**Table S2.** Gate voltage changes of FET devices in the detection of different target-DNA concentrations by using three probes (unit: mV)

| **Identifier** | **Concentration** | | | |
| --- | --- | --- | --- | --- |
|  | **0.1 fM** | **0.5 fM** | **1 fM** | **10 fM** |
| H1-DNA | 26.1±19 | 40.7±34.1 | 78.5±30.5 | 120.1±38.7 |
| H1-nDNAp4 | 86.3±14.6 | 132.8±24.6 | 154.5±6.6 | 206.1±24.7 |
| H1-nDNAp5 | 94.2±28.6 | 142.8±19.6 | 194.6±28.6 | 243±32.6 |

**Table S3.** The values of gate voltage changes and the standard deviation of three blank experiments. The standard error mean of the blank measurements is 11.3 ($19.6/\sqrt{3}$).

|  | **Number of experiment** | | | Standard deviation (σ_reag_) |
| --- | --- | --- | --- | --- |
|  | **1** | **2** | **3** |  |
| **Gate voltage change (mV)** | 4.2 | 3.2 | 37.7 | 19.6 |


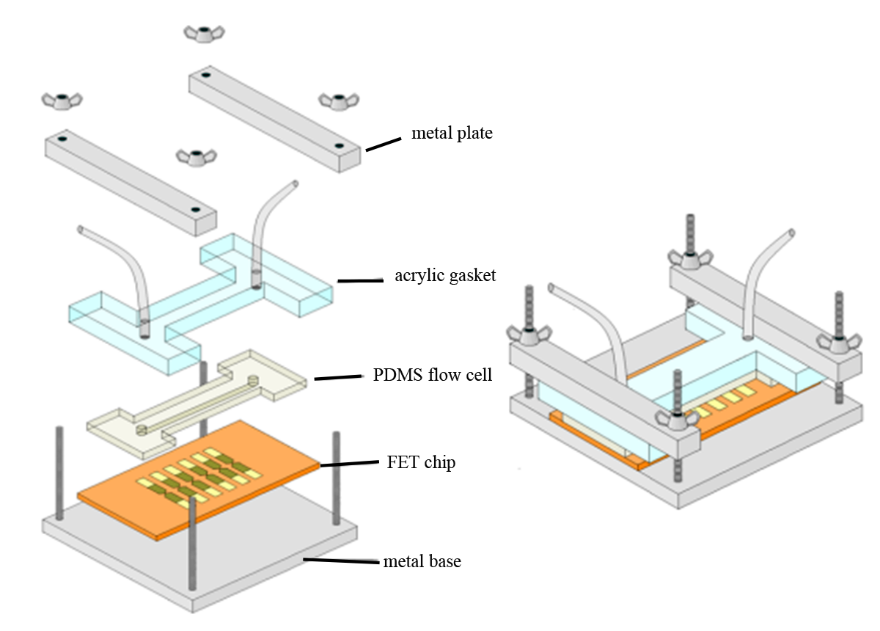


**Fig. S1** The microfluidic system is composed of a PDMS flow cell, an acrylic gasket and metal plates, and the dimension of fluidic channel is 5 × 0.5 × 0.1 mm^3^.

| (a)  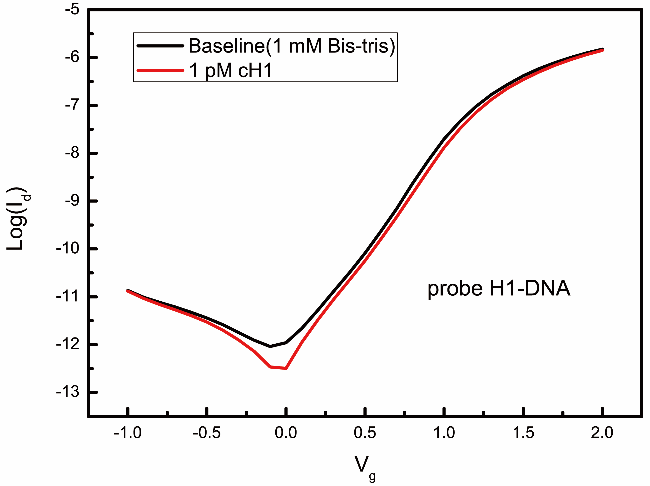 | (b)  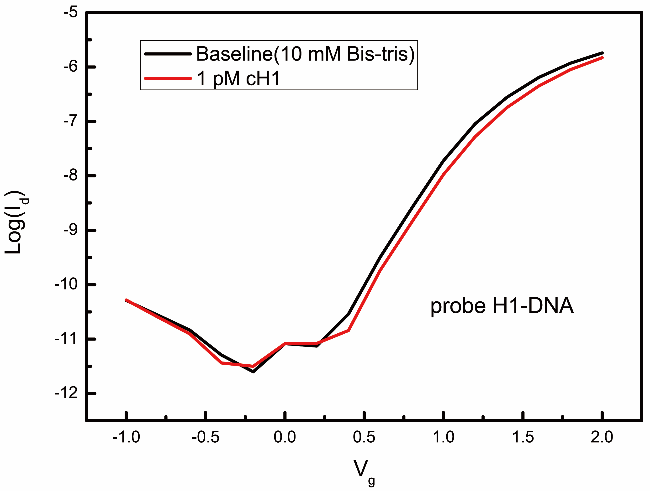 |
| --- | --- |
| (c)  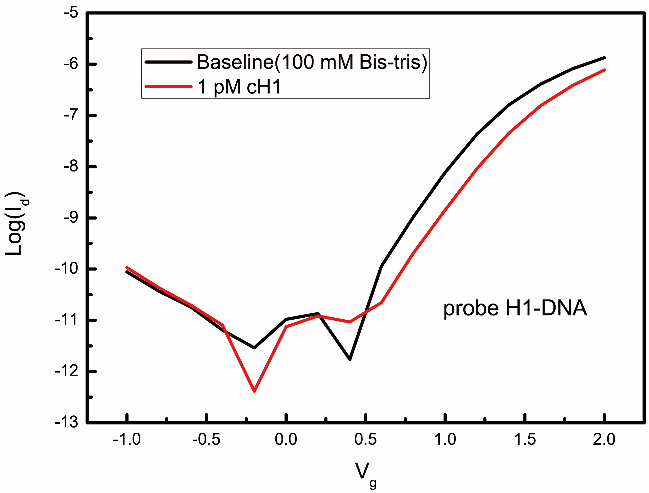 | **(d)**  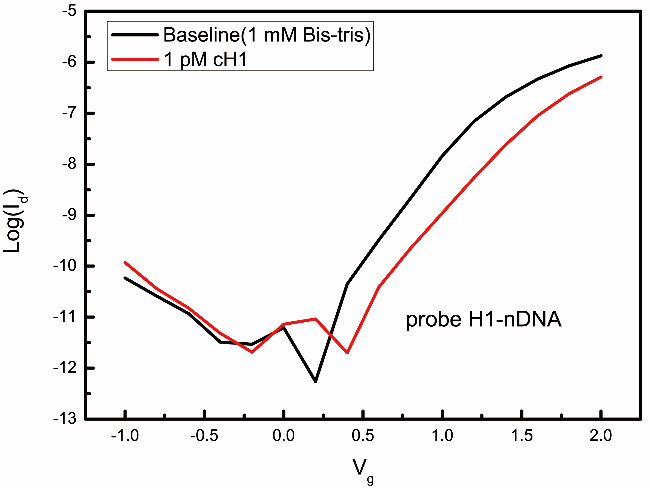 |
| (e)  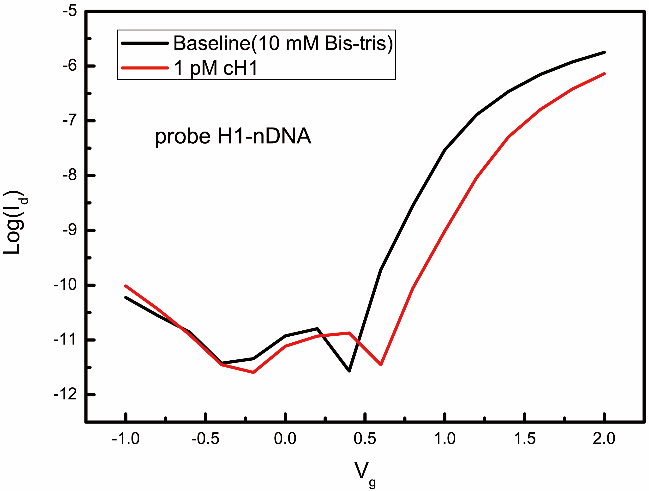 | **(f)**  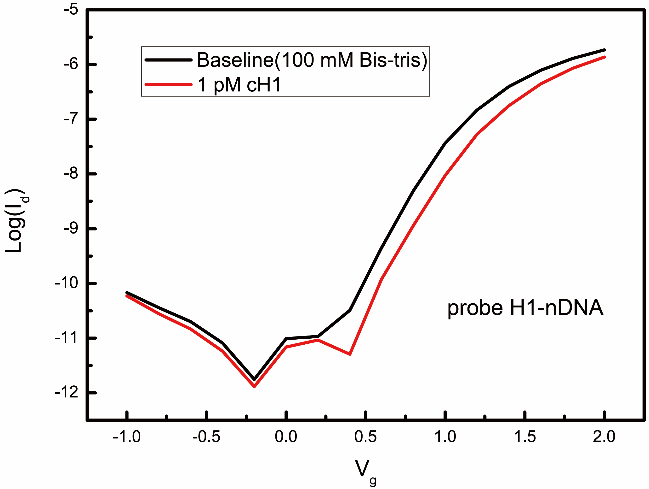 |

**Fig. S2** Current-voltage curves measured on the NWFET devices in 1, 10 and 100 mM Bis-tris propane (BTP) buffers for the detection of target cH1 (1pM) with probes of H1-DNA (a)(b)(c) and H1-nDNA (d)(e)(f).

| (a)  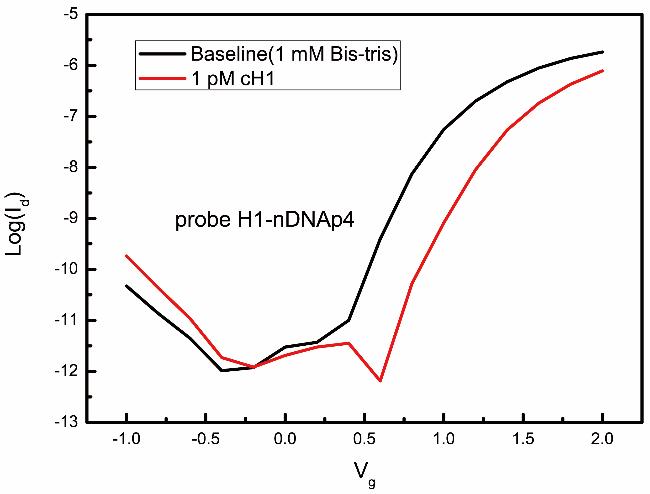 | (b)  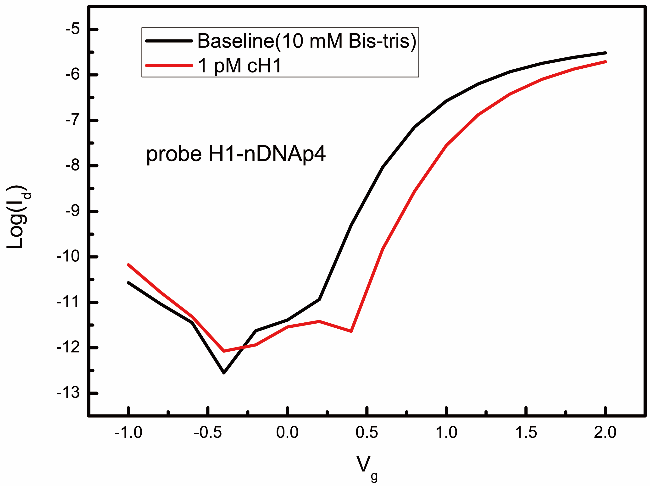 |
| --- | --- |
| (c)  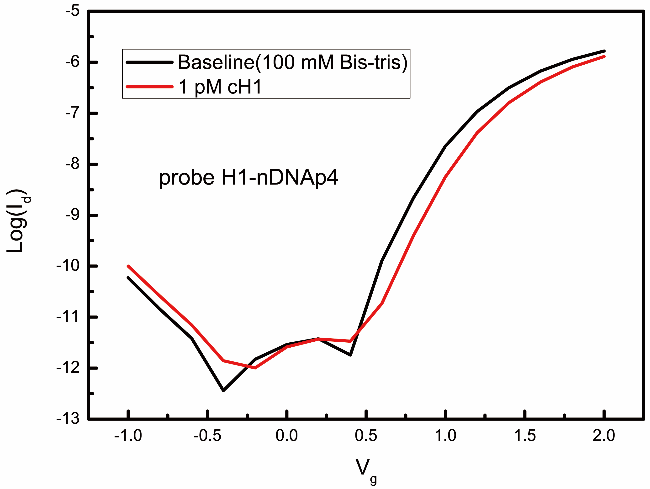 | **(d)**  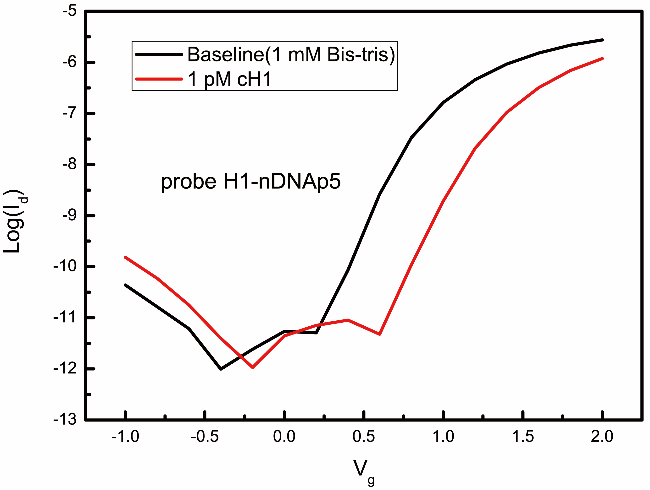 |
| (e)  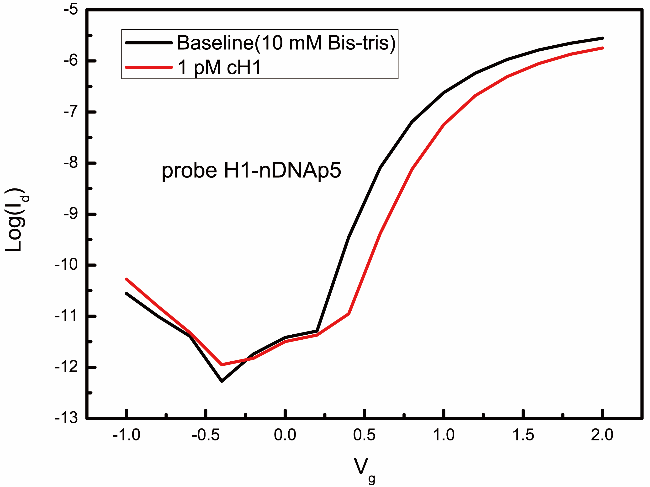 | **(f)**  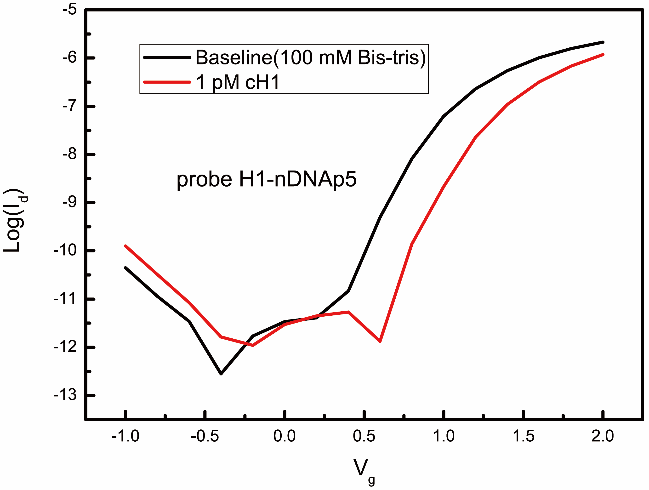 |

**Fig. S3** Current-voltage curves measured on the NWFET devices in 1, 10 and 100 mM Bis-tris propane (BTP) buffers for the detection of target cH1(1pM) with probes of H1-nDNAp4 (a)(b)(c) and H1-nDNAp5 (d)(e)(f).


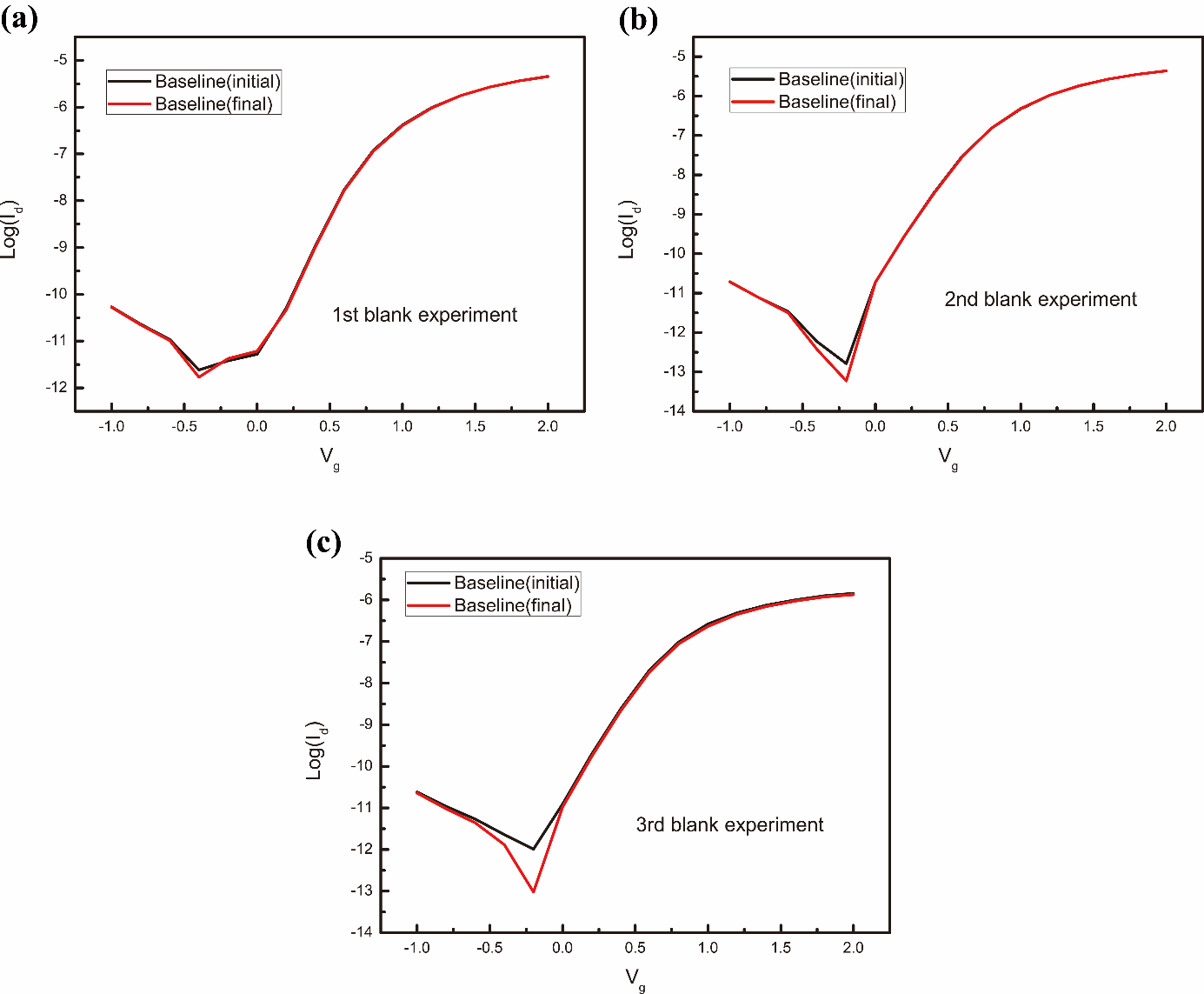


**Fig. S4** The three current-voltage curves of reagent blanks measured in the 1 mM PTB buffer for getting the background signals of the FET devices. (a) 1st blank experiment. (a) 2nd blank experiment. (c) 3rd blank experiment.
